# Psychological Contract, Self-Efficacy, Job Stress, and Turnover Intention: A View of Job Demand-Control-Support Model

**DOI:** 10.3389/fpsyg.2022.868692

**Published:** 2022-05-04

**Authors:** Lijin Shao, Hui Guo, Xiaoyao Yue, Zhaohua Zhang

**Affiliations:** ^1^School of Economics and Management, Fujian College of Water Conservancy and Electric Power, Yonan, China; ^2^Innovation College, North Chiang Mai University, Chiang Mai, Thailand; ^3^College of Teacher Education, Yuxi Normal University, Yuxi, China; ^4^School of Humanities, Jinan University, Zhuhai, China

**Keywords:** job stress, psychological contract, self-efficacy, turnover intention, transformational leadership

## Abstract

The outbreak of Coronavirus disease 2019 (COVID-19) has caused enterprises to face more challenges, such as operational management, production and sales management, and human resource management, among other issues. In the context of the global knowledge economy, employees with high knowledge and skills have become an important source of corporate growth and breakthroughs. However, employees may intend to transfer to other companies due to the pressure of the external and internal environments, so the main topic explored by this paper will be the change of employees' turnover intention. The purpose of this study was to explore the influence mechanism that propels the employees' self-efficacy, job stress, and turnover intention, and the moderating effect of transformational leadership. A total of 553 valid responses from several information service companies in China are collected *via* purposive sampling and used in the data analysis. This study conducts partial least squares structural equation modeling partial least squares structural equation modeling (PLS-SEM) to analyze collected data. The results of the path analysis with structural equation modeling show that employees' psychological contracts have a positive impact on the self-efficacy and a negative impact on the job stress. Employees' self-efficacy has a negative impact on job stress and turnover intention; transformational leadership plays a significant moderator in the research framework. Based on research findings, the theoretical and managerial implications are presented.

## Introduction

The Coronavirus disease 2019 (COVID-19) pandemic has dramatically hit economic and social operations around the world, resulting in a diversity of risks and challenges facing organizations (Lee et al., 2021; Said et al., 2021). In particular, organizations in countries with transition economies find themselves confronted by more business distress, such as declining market shares, lack of innovation capability, reduced capital liquidity, and increased staff turnover (Vu et al., 2019; St-Jean and Duhamel, 2020). Affected by COVID-19, many organizations cannot provide suitable working environments, and even the factory production ceases, so the turnover intention may come about when the job demands cannot be satisfied. In view of the knowledge base, employees are the source of organizational competitiveness. The decline of the staff turnover or turnover intention will be conducive to improving other negative factors (Urbancová and Linhartová, 2011; De Silva et al., 2018). Specifically, the turnover intention refers to the attitude of employees about leaving or changing companies they served because of uncertainties generated when they face individual or organizational factors (Urbancová and Linhartová, 2011; De Simone et al., 2018; Moquin et al., 2019). Previous studies showed that the determinant of turnover intention has multiple dimensions such as organizational & individual, internal & external, and positive & negative factors. The majority of studies offered their insights and contributions with the purpose of improving or decreasing the turnover intention of employees (Park and Jung, 2015; Afzal et al., 2019). Combined with the job demand-control (JDC) (support) model (Beehr and McGrath, 1992; Taylor et al., 1997), this study presents a complete model to discuss the turnover intention of employees from the positive and negative perspectives.

As shown in the literature, indicators of employees' turnover intentions are not only correlated with the individual attributes (Park and Jung, 2015; Albrecht and Marty, 2020), but also depend on the work context (De Simone et al., 2018). The work context can be defined as the dynamic or static group characteristics and organizational attributes of companies/organizations where employees serve (Albrecht and Marty, 2020; Khan et al., 2021). Examples include two-way communication and supervisor leadership, organizational resources and encouragement, or organizational support and cultural climate, and other management practices. Furthermore, the employment pattern of companies/organizations include features by which organizations are able to understand the career development expectations of employees, and enable employees to satisfy agreed rights and interests promised by organizations (Moquin et al., 2019; Boudrias et al., 2020); employees, in turn, contribute their expertise for the long-term development of organizations based on their trust that the organizations will actively perform the work contract (Moquin et al., 2019; Albrecht and Marty, 2020). The original working conditions of employees were changed due to COVID-19, bringing concerns about losing their jobs, causing employees to seek alternatives to avoid the shock of losing their jobs. In short, employees' psychological contract to organizations is an important influence factor for the turnover intention (Chen and Wu, 2017; Duran et al., 2019; Khan et al., 2021). However, as stated in previous literature on the psychological contract, the effectiveness of the psychological contract can be elevated only when there are some mediating variables between the psychological contract and outcome variables (Hartmann and Rutherford, 2015; Said et al., 2021). Thus, this study aims to explore the relationship between psychological contract and turnover intention, and the effect of mediating variables between them.

Based on the JDC (support) model, the higher job control and job decision latitude given to employees can relieve the job strain generated from job demands and facilitate their learning motivation (Bruyneel et al., 2017; Vassos et al., 2019). On the whole, this model holds that the context forming based on different combinations of job demands and job control can affect the job strain and learning in different ways. Specifically, the context with higher requirements and lower control can lead to a higher degree of job strain (Ohlson et al., 2001), which is called “stress hypothesis,” while the context with higher requirements and higher control will facilitate the learning, which is called “positive hypothesis.” Theorell et al. (1990) further pointed out that positive context and successful learning opportunities can increase the productivity of employees by enhancing their confidence and feeling of competence (Ohlson et al., 2001; Ariza-Montes et al., 2018). In addition, job control can also relieve the negative effects caused by higher job demands, which is called “cushion hypothesis” The turnover intention of employees can also be affected by job strain and job control. This means the discussion of employees' turnover intention from the perspective of positive and negative psychological factors will be contributive to building a more complete model (Vassos et al., 2019; Boudrias et al., 2020). The positive psychological factors depend on the control of employees over the job. This is consistent with the self-efficacy theory that believes a higher level of employee belief in finishing the work will promote the control over job execution and reduce uncertainties for the job (Park and Jung, 2015; Boudrias et al., 2020). The negative psychological factors are highly correlated with job strain and job stress. A higher degree of job strain may produce a higher degree of job stress, and vice versa (Chung et al., 2017; Park et al., 2020). The job stress is considered as a factor that is favorable to the improvement of work efficiency in different studies. Appropriate job stress allows employees to feel the urgency of job execution, thus leading to a higher performance. However, excessive stress can induce negative physical and psychological status, such as lower wellbeing, anxiety, and higher turnover intention (Chung et al., 2017; Ariza-Montes et al., 2018; Wen et al., 2020). Based on the above statements, this study aims to discuss the effect of self-efficacy and job stress on turnover intention, and verifies the mediating role of them in psychological contract and turnover intention.

Scholars indicated that the JDC model is oversimplified, because the job control is the only one of many resources for coping with job stress, and overlooks the equally important social support (Ariza-Montes et al., 2018). COVID-19, as a new topic, will have a different impact on employees' job stress compared to the results of previous studies, and the discussion from this perspective will be conducive to verifying old theoretical models and offering meaningful insights. The social support has always been regarded as having an immediate and interactive effect on job strain and the psychological and physical health of employees (Pozo-Antúnez et al., 2018). Johnson and Hall (1988) expanded the JDC model as the JDC-Support (JDCS) model by introducing the social support (Bruyneel et al., 2017; Vassos et al., 2019). The JDCS model emphasizes that organizations or leaders will give tangible and intangible resources based on the working situation of employees, and assist employees to effectively control the operation of work tasks and their psychological stress (Pozo-Antúnez et al., 2018). This study presents that the leadership relationship between supervisors and employees can be regarded as an important support factor, and the differences in the leadership style will result in an obvious contrast in the work efficiency of employees (Lornudd et al., 2015). Specifically, the transformation leadership is an important source of support, which can enlighten and guide employees to generate a high level of organizational citizenship behaviors (Zhang et al., 2018) and arouse the high sense of identity among employees together with other factors, thus bringing down the turnover intention (Sun et al., 2012; Arnold and Walsh, 2015; Nohe and Hertel, 2017). Thus, this study aims to explore the mediating role of the transformation leadership of supervisors in the research model.

Based on the above statements, this study makes several contributions, such as (1) verifying the impact of psychological contract on turnover intention using the (JDCS) model and deepening the understanding of psychological contract, (2) changing the status that all previous studies take positive factors as an important source to discuss the job demands of employees by taking positive and negative factors and control factors, and (3) considering transformation leadership as important modulating culture in organizations, discussing relationships between variables and enhancing the model diversity.

## Literature Review and Hypotheses Development

### Job Demand-Control-Support Model

Proposed by Karasek (1979), the JDC model points out that the joint effect of job demand and job strain will affect the job strain (Lornudd et al., 2015; Ariza-Montes et al., 2018). The job demand is the psychological source of stress, such as work overload, time stress, and conflict demands, and the job decision latitude refers to the decision-making authorization and decision-making power in terms of technologies gained by employees. Many recent studies combined the two dimensions and changed the name of job decision latitude to job control (Theorell et al., 1990; Brough et al., 2018). Johnson and Hall (1988) expanded the JDC model as the JDCS model by introducing the social support; however, the social support has always been regarded having an immediate and interactive effect on work strain and the psychological and physical health of employees (Bruyneel et al., 2017; Brough et al., 2018; Vassos et al., 2019). Meanwhile, under the high job demands, what employees worry about most is whether they can finish the work smoothly, and the support and affirmation from supervisors and colleagues can provide a relieved work environment with positive feedback (Ariza-Montes et al., 2018). It thus appears that the social support is beneficial to enhance the employees' confidence in completing the work and the perception of mutual concern (Pozo-Antúnez et al., 2018). In order to clearly understand the determinants of the turnover intention under the COVID-19 pandemic, this study, based on the JDCS model, expects to offer richer insights into the theoretical basis, with the psychological contract as the independent variable, the self-efficacy and job stress as mediating variables, and the transformation leadership style of supervisors as the disturbance variable.

### Job Stress

Selye (1956) classified the stress sources into eustress and distress. Stress sources of different natures vary in motivational potency, and will lead to different outcome efficacies (Ohlson et al., 2001). Wen et al. (2020) defined the job stress as a process where individual psychological and physiological status changes under the interactive effect of job-related factors and the job. The job stress can also be defined as the reflection of workers' failures in adapting to the work environment (Chung et al., 2017), and can be classified into interpersonal relationship stress, professional knowledge stress, workload stress, self-expectation stress, organizational change stress, etc. (Park et al., 2020; Wen et al., 2020). It will have a direct impact on workers, and will even cause physical, psychological, and behavioral changes of individuals (Akgunduz and Gürel, 2019). Previous studies of job stress focused on of discussing the effect of specific stress sources such as role conflict and role ambiguity on job stress (Sethi et al., 1999). However, the job stress does not only derive from role conflict and role ambiguity, but also from some other factors such as workload, leadership style of supervisors, and worktime urgency, so any source resulting in the psychological stress of individuals can also be included in the discussion (Brough et al., 2018; Wen et al., 2020). Cavanaugh et al. (2000) argued that individuals judge whether the stress source can be controlled based on their perception of whether this stress is affected by a controllable cognition; as a result, individuals with the ability to solve the stress source will see this an opportunity for growth. On the contrary, if some stress sources are considered uncontrollable or unsolved, individuals will be impeded from seeking opportunities for growth (Park et al., 2020).

The job stress of employees is an important subject, which not only causes a negative impact on employees, but also brings damages to employers (Akgunduz and Gürel, 2019; Wen et al., 2020). Some scholars found that the role of stress arising from job stress is positively correlated with the turnover intention of employees (Akgunduz and Gürel, 2019; Wen et al., 2020). Some other scholars indicated that the reduction of job stress can reduce the emotional exhaustion of employees (Isaksson Ro et al., 2010). It is also found that job stress has a negative correlation with job performance, work ethic, and job satisfaction (Kalyar et al., 2019), and a positive correlation with job burnout and turnover intention (Hwang et al., 2014; Chiang and Liu, 2017; Chung et al., 2017). If the perceived job stress of employees is self-evaluated as unsolved or as an obstruction to development, employees will be aware of threats from the stress, and may have responses such as worry, anxiety, and fear (Chung et al., 2017; Sahin and Çetin, 2017; Park et al., 2020), resulting in the lack of motivation required to satisfy job demands, the intention of retreating from the job, and even the turnover intention (Akgunduz and Gürel, 2019; Park et al., 2020; Wen et al., 2020). Thus, this study develops a hypothesis as follows:

H1: Job stress has a positive impact on turnover intention.

### Self-Efficacy

Self-efficacy refers to one's belief in individual capabilities to perform well in specified work at a designated level (Bandura, 1986; Ugwu and Oji, 2013). As a self-regulatory mechanism (Guarnaccia et al., 2018) or personal resource (Hobfoll, 2001), self-efficacy has been given the role of mediator to manage employees' perceptions of job dissatisfaction or job insecurity (Shih and Chuang, 2013; Etehadi and Karatepe, 2019; Van Hootegem and De Witte, 2019), which influence the employees' service innovation behaviors (Kim et al., 2018a; Etehadi and Karatepe, 2019). In the present study, self-efficacy can be used to predict the employees' performances related to family and social aspects. Ugwu and Oji (2013) proposed that individuals with high self-efficacy showed a higher tendency of prosocial behaviors. In addition, self-efficacy has an impact on emotional reactions (Gist and Mitchell, 1992; Karatepe, 2015; Etehadi and Karatepe, 2019), so that self-efficacy can be a solution to improve the ability of employees to successfully respond to related disappointments with an organization that has not fulfilled commitments (De Clercq et al., 2020). Based on the diverse research contexts, researchers have extended a variety of self-efficacy to identify the employees' outcomes under different situations, such as knowledge sharing self-efficacy (Ye et al., 2015), occupational self-efficacy (Rigotti et al., 2008), and work-related self-efficacy (Bandura, 1997; Schmitz and Ganesan, 2014; Dechawatanapaisal, 2018).

Compared to employees with a low self-efficacy, those with a high self-efficacy clearly understand to what extent they can finish tasks under specific social contexts (Sahin and Çetin, 2017; Afzal et al., 2019). Employees with a high self-efficacy will be more focused and make greater efforts in the face of contexts or statuses, and will exude confidence (Albrecht and Marty, 2020). Meanwhile, they will also make greater efforts to try to make breakthroughs and persist, thus reducing the risk and uncertainty caused by job fatigue or job stress (Brough et al., 2018; De Simone et al., 2018; Afzal et al., 2019). It can thus be seen that self-efficacy will be a critical and significant indicator when discussing how to reduce the negative emotion and attitudes caused by job stress (Sahin and Çetin, 2017). As scholars have indicated, employees with a low self-efficacy may easily retreat in the face of difficulties and lack specific goals because of insufficient concentration and unclear directions (De Clercq et al., 2018); this kind of model will spend more time on and devote more thought to the uncertain attempts, resulting in a higher level of job stress. Thus, this study proposes a hypothesis as follows:

H2: Self-efficacy has a negative impact on job stress (Park et al., 2020).

Employees with a higher level of self-efficacy are willing to set high goals, are not afraid of failure, and are more likely to persist until difficulties are overcome (Khan et al., 2021). On the contrary, employees with a lower level of self-efficacy are not willing to put in practice, and are more likely to give up in the face of difficulties. As stated by scholars, if employees have a higher sense of efficacy for their future, they will have a healthier psychological and physical status, and develop high expectations and achievement motivations for themselves, thus resulting in reduced feelings of discouragement (Park and Jung, 2015; De Simone et al., 2018). Some other studies also indicated that the employees with a lower level of self-efficacy are less responsible for dealing with challenges, while those with a higher level of self-efficacy are highly motivated to cope with challenges or achieve innovation goals, thus resulting in good performances and reduced turnover intention (Afzal et al., 2019; Albrecht and Marty, 2020). Thus, this study proposes a hypothesis as follows:

H3: Self-efficacy has a negative impact on turnover intentions.

### Psychological Contract

Psychological contract refers to an unwritten contract with mutual and implicit expectations between employee and organization (Levinson et al., 1962), or beliefs of an employee about reciprocal and promissory but not recognized obligations, which are based on the perceived promises between employees and employers or organization (Rousseau, 1990; Morrison and Robinson, 1997). Current studies use the psychological contract to identify the employees' sense that employer or organization has breached the contract (Ugwu and Oji, 2013), leading to the employee's feeling of betrayal and psychological suffering in response to broken promises and unmet expectations (Robinson and Wolfe Morrison, 2000), which refers to psychological contract violation or psychological contract breach. In most of these studies, researchers use the psychological contract as a framework to understand the employees' attitude or behavior (Coyle-Shapiro and Kessler, 2003), and a potential solution to the acquisition and retention of employees (Lub et al., 2012; Lu et al., 2016). However, Van Hootegem and De Witte (2019) considered psychological contract breach as a mediator rather than a framework, to understand the relationship between job insecurity and informal learning. As well as focusing on formal employees and discussion in the context of profitable organizations, the psychological contract is also used to identify the influence of the psychological contract between companies and users in an online community (Liu et al., 2021), and in government organizations to identify workers' perceptions and emotions, for example, Duran et al. (2019) proposed that psychological contract violation could lead to job-related stress of firefighters. Now, scholars continue to enrich the theory of psychological contract according to its subjective and idiosyncratic nature (Rousseau and Tijoriwala, 1998), involving individual traits combined to predict psychological contract, while Shih and Chuang (2013) use narrow traits and broad traits to find individual differences, influencing the mediation relationship between psychological contract breach and other aspects.

The psychological contract shows that individuals have a close and long-term relationship and norm with organizations, which strongly affect the individuals' professional career development and advancement (Hartmann and Rutherford, 2015; Kim et al., 2018b). In addition, the psychological contract can also be considered as a process of socialization of individuals in organizations (Birtch et al., 2015). The exchange relationship between employees and organizations gradually extends from the performance of the formal contract to the identification with informal norms and obligations (Chen and Wu, 2017; Said et al., 2021). If employees clearly know their responsibilities and work scopes and further enhance the identification and commitment with organizations, they will have a better understanding of the vision, mission, and goals of organizations (Lu et al., 2016; Liu et al., 2021), which will drive them to enhance their professional skills to finish tasks assigned by organizations (Duran et al., 2019). Scholars have indicated that the high level of identification with organizations will facilitate employees to elevate their beliefs and attitudes required to accomplish tasks and achieve the goals shared by organizations and employees. Thus, this study proposes a hypothesis as follows:

H3: Psychological contract has a positive impact on self-efficacy (Kim et al., 2018b).

The majority of job stress derives from unfamiliarity with job contents and scopes of organizations, and excessive workloads may make it difficult for employees to absorb the negative attitudes resulting from their work or their lives (Duran et al., 2019). As indicated by scholars, an appropriate level of job stress allows employees to improve performance or gain more advantageous creative ideas, or a low level of job stress can reduce the turnover intention of employees. But they have neglected the question of how to make employees recognize the level of appropriate stress (Birtch et al., 2015; Duran et al., 2019). Although few studies have discussed this topic, a better understanding of formal and informal norms and a higher level of identification of employees will help them have a clearer grasp of job contents (Birtch et al., 2015; Hartmann and Rutherford, 2015). A high level of job stress may result from employees' insufficient cognition of organizational demands, leading to difficulties in providing effective solutions or satisfying the demands required for completing the job and tasks through the development of professional skills (Hartmann and Rutherford, 2015). Employees with a high level of psychological contract are able to identify conditions required to complete tasks, thus further reducing the job stress arising from uncertainties of job tasks. Thus, this study proposes a hypothesis as follows:

H4: Psychological contract has a negative impact on job stress.

### Transformation Leadership

According to the transformation leadership theory, leaders motivate subordinates by promoting their goals and individual care, thus enhancing their self-confidence and enabling them to achieve the performance that exceeds the norm (Basu and Green, 1997; Ramsey et al., 2017). For example, employees who can perceive the transformation leadership of supervisors at a higher level are more able to present innovation behaviors; or when supervisors present the moral modeling or individualized consideration under the transformation leadership, a positive effect will also be exerted on the formation of employee innovation (Li et al., 2015). Besides, the transformation leadership can also positively facilitate employees to improve their innovation behaviors or creative performance through mediating mechanisms such as psychological empowerment of employees (Sun et al., 2012), creative self-efficacy (Gong et al., 2009), and intrinsic motivation (Shin and Zhou, 2003). Since employees are members of job teams led by supervisors, this study states that leadership behaviors presented by leaders should be regarded as an overall leadership style (Vasilaki et al., 2016), which is shared by all team members (Arnold and Walsh, 2015). Thus, this study considers the research level of transformation leadership as a kind of overall leadership behavior produced and dispersed in the workplace, forming a kind of ambient stimulus, i.e., a shared function (Liao and Chuang, 2007; Zhang et al., 2018). For example, the transformation leadership features the emphasis on self-values, beliefs, and missions to create a shared vision, and the formation of employees' identification with roles of leaders in a flexible manner to form the cohesion among employees.

As stated by views on interaction, individual behaviors and attitudes are the outcome of the interaction of characteristics and situations (Arnold and Walsh, 2015). Mediators may affect individual attitudes and behaviors by interacting with other situations or contexts (Stam et al., 2010). Gorman et al. (2012) suggested that future studies should take situations and contexts such as leadership style and task characteristics into account (Wallace et al., 2013). Some other scholars also held the same view (Dimotakis et al., 2012; Sacramento et al., 2013). Previous studies of organizational behaviors have shown that leader behaviors play a significant role in affecting the interactive status and outcomes of employees in organizations (Wang et al., 2013), and the transformation leadership can be considered as a situational factor. Transformation leadership makes greater efforts in motivating subordinates to achieve job goals through performance. Such leaders are adept at stimulating employees to make innovation and changes by making full use of powers and situations (Zhang et al., 2018). They are also willing to delegate powers to subordinates, and to help subordinates release potential abilities to achieve organizational goals (Arnold and Walsh, 2015). Besides, as stated in a few studies, transformation leadership is contributive to enhancing the positive psychological factors of employees and reducing the turnover intention. The support of supervisors will be beneficial to the building of trust between the employees and supervisors, and developing the attitude of reciprocity in employees' psychological contract, thus forming abilities and beliefs required to complete the task goals and reducing the possibility of causing damages by job stress (Ayoko and Callan, 2010; Vasilaki, 2011; Vasilaki et al., 2016). In addition, in the process of social identification, transformation leadership can affect their followers through taking the self-concept of followers into account (Vasilaki et al., 2016), and exert influence on attitudes or performances of team members and individual employees (Zhang et al., 2018) to reduce the turnover intention. Thus, the inspirational motivation of transformation leadership will motivate employees to grow and to share visions and ideas, strengthening more positive outcomes such as self-efficacy. Intellectual stimulation encourages employees to figure out diversified ideas and new approaches to solve problems, and stimulates deeper development and more positive development (e.g., reducing job stress) (Miao and Cao, 2019). Based on the above statements, we can see that the transformation leadership is a situational driver for achievement of goals. Thus, we develop hypotheses as follows:

H6: Transformational leadership moderates relationship between psychological contract and self-efficacy.
H7: Transformational leadership moderates relationship between psychological contract and job stress.
H8: Transformational leadership moderates relationship between self-efficacy and turnover intention.
H9: Transformational leadership moderates relationship between job stress and turnover intention.

**Figure 1:**
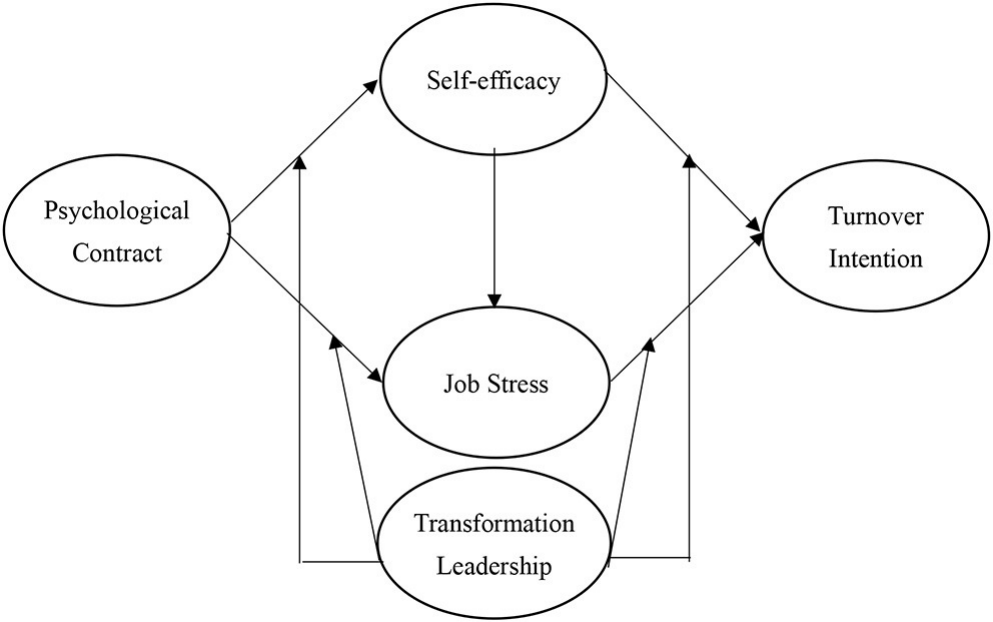
Research framework.

According to the above hypotheses, the research framework is shown in Figure 1.

## Methodology

### Sampling

This study aims to understand the psychological characteristics of front-line staff (which refers to employees who interact and communicate with customers face to face and provide service required by customers) during major events, especially during the COVID-19 pandemic. It explores the relationship between perceived organizational support, subjective wellbeing, and job performance. As there are different quarantine procedures in different countries, and the pandemic plays different influences on the people's psychological characteristics, it is impracticable to take each country as a sample. However, employees in industries of different natures differ greatly in job attributes and properties. Furthermore, it is difficult to collect samples by random sampling due to the huge number of employees in the Chinese mainland. In order to enhance the sample representativeness, researchers select effective sample clusters based on their research purposes and issues. Thus, purposive sampling is adopted, and several conditions will be established during sampling so as to improve the representativeness of the research samples. First, mainland China, where the pandemic was most severe in the beginning, was selected as the main area for sampling, and the quarantine policy was the strictest. Thus, it is representative to a certain extent. Second, to understand the psychological characteristics of front-line staff, it is necessary to focus on those who actually face customers, and the service industry was adopted as the main industry. Third, while filling the questionnaire, all the samples were already at work, rather than being isolated at home. This study takes the front-line staff in the service industry, excluding the staff in the catering service industry, as the study population in order to accurately collect representative samples. In this study, copies of the electronic questionnaire were sent, and 582 copies of the questionnaire were collected. Five hundred and fifty three copies of the valid questionnaire were obtained after excluding invalid ones. In the sample, most are male (58.3%).

### Measures

The psychological contract adopted the scale revised by Kraak et al. (2017), which owns six measuring dimensions of job content, career development, social atmosphere, organizational policies, work-life balance, and rewards, as well as 21 measuring items, such as “offer possibilities for good cooperation,” “professional development opportunities,” and “clear and fair rules and regulations.” The Likert five-point scale was generally used with 1 (strongly disagree) to 5 (strongly agree).

For Self-efficacy, the scale revised by Alisic and Wiese (2020) was adopted, and it was revised to integrate 3 items of higher reliability and validity, such as “I can remain calm when facing difficulties in my job because I can rely on my abilities” and “My past experiences in my job have prepared me well for my occupational future.” Likert five-point scale was generally used with 1 (strongly disagree) to 5 (strongly agree).

Job stress adopted the scale revised by Tongchaiprasit and Ariyabuddhiphongs (2016), which owns two measuring dimensions of workload and insufficient resources, as well as 13 measuring items, such as “lack of feedback on performance,” “insufficient management support,” and “poor communication between staff.”

Turnover intention adopted the scale revised by Dane and Brummel (2014), and it was revised to integrate 4 items of higher reliability and validity, such as “I am thinking about leaving this organization” and “I intend to ask people about new job opportunities.”

In this study, we adapt the multi-item scale modified from the transformation leadership Index on six aspects of transformation leadership dimensions proposed by Ramsey et al. (2017), also well-known as bundle of transformation leadership. Transformation leadership was measured by six dimensions, such as identifying and articulating a vision (5 items), providing an appropriate model (3 items), fostering the acceptance of group goals (4 items), high performance expectations (3 items), providing individualized support (4 items), and intellectual stimulation (4 items).

## Results

### Assessment of Measurement Model

This study evaluates and revises the CFA measurement model based on a two-stage model (Kline, 2011). Currently, academics generally agree with the approach of Anderson and Gerbing (1988). That is, CFA should report Standardized Factor Loading, Multivariate Correlation Squared, Composite Reliability, and Average Variance Extracted for all variables, and only after these metrics pass the test can structural models be evaluated. Specifically, Standardized Factor Loading is >0.50, Composite Reliability is >0.60, and Average Variance Extracted is >0.50 (Hair et al., 2017), then the measurement model has good convergent validity. Table 1 reports the CFA of the measurement models, indicating that each construct has good convergent validity. Discriminant validity is a measure to test whether any two variables in a theoretical model are identical to each other. The square root of AVE for each latent construct (see Table 1) is greater than its cross-correlation with other constructs, confirming discriminant validity.

**Table 1:** Measurement.

	**1**	**2**	**3**	**4**	**5**	**6**	**7**	**8**	**9**	**10**	**11**	**12**	**13**	**14**	**15**	**16**
1. JC	0.880															
2. CD	0.832	0.900														
3. SA	0.734	0.852	0.949													
4. OP	0.753	0.828	0.871	0.883												
5. WLB	0.590	0.623	0.629	0.716	0.849											
6. Rewards	0.677	0.756	0.771	0.800	0.727	0.897										
7. Self-efficacy	0.702	0.688	0.652	0.687	0.513	0.609	0.912									
8. Workload	0.209	0.200	0.188	0.237	0.317	0.243	0.210	0.803								
9. IR	0.074	0.089	0.073	0.100	0.258	0.170	0.099	0.781	0.804							
10. TI	−0.122	−0.137	−0.138	−0.112	0.064	−0.054	−0.108	0.498	0.557	0.945						
11. IAV	0.527	0.467	0.387	0.424	0.325	0.323	0.529	0.036	−0.025	−0.162	0.868					
12. PAM	0.521	0.467	0.388	0.414	0.298	0.311	0.500	0.020	−0.058	−0.191	0.831	0.939				
13. FAGG	0.538	0.491	0.413	0.423	0.307	0.344	0.541	0.017	−0.055	−0.198	0.822	0.864	0.940			
14. HPE	0.562	0.549	0.469	0.521	0.421	0.449	0.538	0.058	−0.027	−0.175	0.738	0.741	0.788	0.853		
15. PIS	0.465	0.379	0.310	0.331	0.293	0.238	0.435	0.019	−0.022	−0.144	0.723	0.716	0.702	0.635	0.892	
16. IS	0.550	0.495	0.406	0.424	0.346	0.357	0.513	0.057	−0.013	−0.149	0.764	0.763	0.792	0.745	0.755	0.926
Cronbach's α	0.854	0.941	0.889	0.929	0.809	0.878	0.898	0.889	0.908	0.960	0.918	0.933	0.938	0.814	0.914	0.917
AVE	0.774	0.810	0.900	0.779	0.720	0.805	0.831	0.645	0.646	0.893	0.753	0.882	0.884	0.728	0.796	0.858
CR	0.911	0.955	0.947	0.946	0.885	0.925	0.937	0.916	0.927	0.971	0.938	0.957	0.956	0.889	0.940	0.948

### Hypotheses Testing

**Figure 2:**
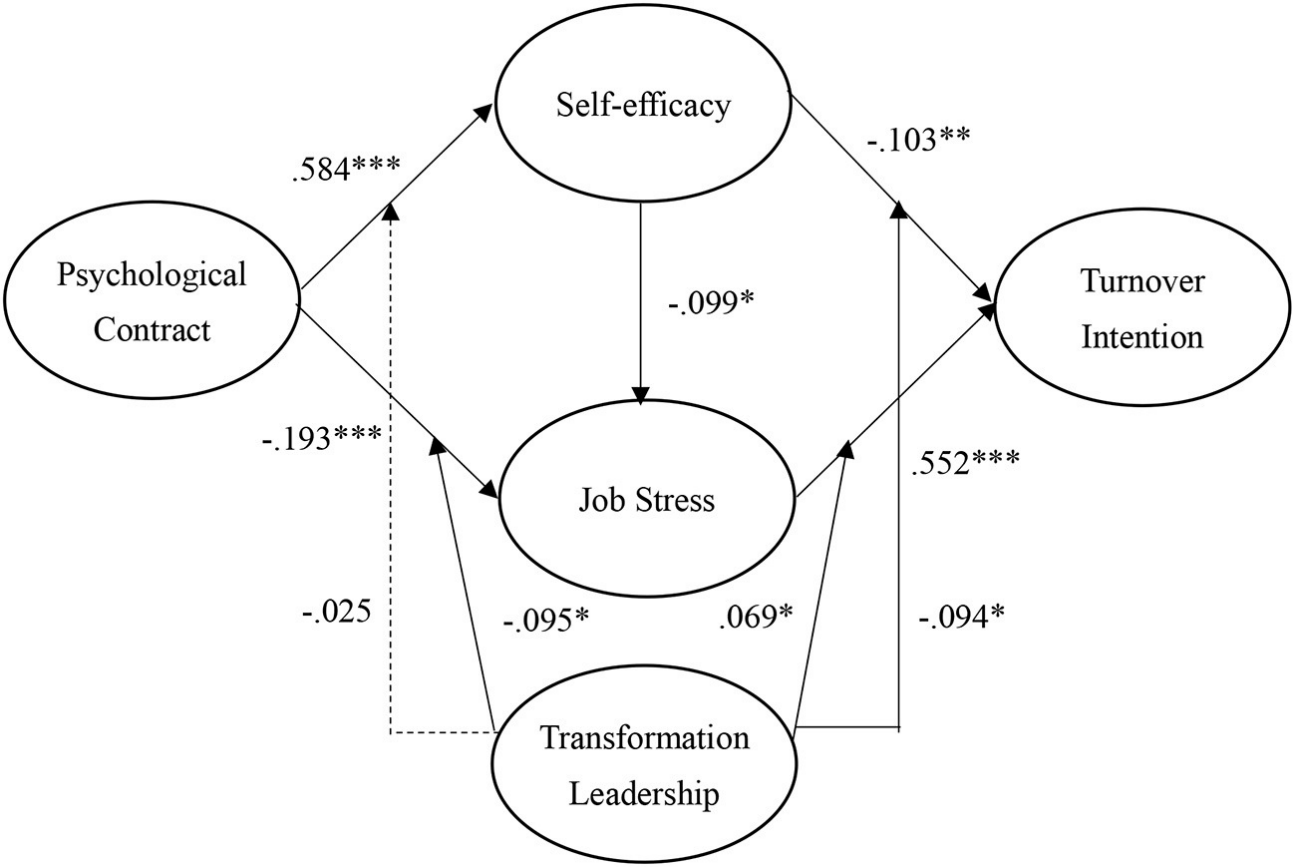
Structural model.

This study was adopted PLS-SEM to represent the structural regression coefficients and explained variance in the endogenous latent variables. According to Hair et al. (2017), the bootstrapping approach is used with a resampling of 5,000 for evaluating the significance of the path coefficient. As shown in Figure 2 and Table 2, results show the standardized regression coefficients of the direct effects between the latent variables.

**Table 2:** Results of hypotheses testing.

**Paths**	**Coefficients**	***t*-value**	**Results**
H1: Job stress → Turnover intention	0.552	13.403	Confirmed
H2: Self-efficacy → Turnover intention	−0.103	2.231	Confirmed
H3: Self-efficacy → Job stress	−0.099	2.113	Confirmed
H4: Psychological contract → Self-efficacy	0.584	9.987	Confirmed
H5: Psychological contract → Job stress	−0.193	3.392	Confirmed
H6: Psychological contract*Transformational leadership → Self-efficacy	−0.025	0.605	Not confirmed
H7: Psychological contract*Transformational leadership → Job stress	−0.095	1.983	Confirmed
H8: Self-efficacy*Transformational leadership → Turnover intention	0.069	1.973	Confirmed
H9: Job stress*Transformational leadership → Turnover intention	−0.094	2.065	Confirmed

As displayed in Figure 2 and Table 2, the findings indicate that the job stress (β = 0.246, *p* < 0.001) is positively correlated with turnover intention, which supports H1. Moreover, self-efficacy is negatively and significantly correlated with job stress (β = 0.246, *p* < 0.001) and turnover intention (β = 0.246, *p* < 0.001), respectively; H2 and H3 are supported. Similarly, psychological contract has a positive impact on self-efficacy (β = 0.246, *p* < 0.001) and a negative impact on job stress (β = 0.246, *p* < 0.001), so H4 and H5 are confirmed. Our findings also evidence that transformational leadership significantly moderates the relationships of psychological contract to self-efficacy (β = 0.246, *p* < 0.001), psychological contract to job stress (β = 0.246, *p* < 0.001), self-efficacy to turnover intention (β = 0.246, *p* < 0.001) and job stress to turnover intention (β = 0.246, *p* < 0.001); thus, H7, H8, and H9 are supported rather than H6.

**Figure 3:**
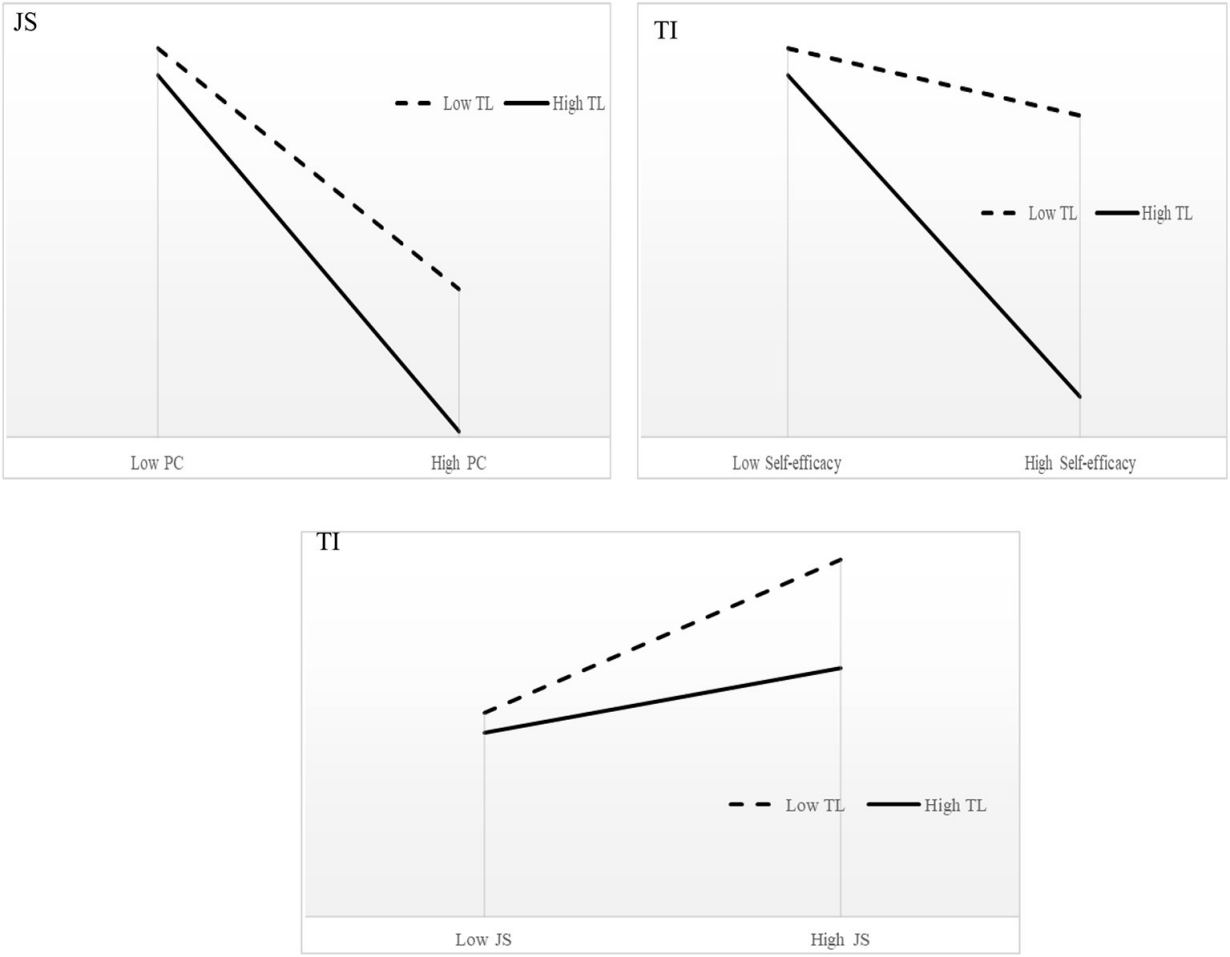
Interaction effects. PC, psychological contract; TL, transformational leadership; TI, turnover intention; JS, job stress.

The interactions among transformational leadership, psychological contract, self-efficacy, and job stress are significant for self-efficacy, job stress, and turnover intention. To show the moderating effects among these relationships more clearly, we plotted these significant interactions and indicated that psychological contract, self-efficacy, and job stress significantly predict the employees' self-efficacy, job stress, and turnover intention only when their transformational leadership is high, as shown in the simple slope chart in Figure 3.

## Conclusions

### Discussion

In the global COVID-19 pandemic, many organizations have introduced human resource management resource practices in terms of improving working conditions for employees and enhancing their job security in order to reduce the turnover intention and mobility. A lot of literatures have discussed the relationship between job stress and performance, as well as the relationship between job stress and turnover intention. However, few studies have been conducted on employee perceived self-abilities from the perspective of psychological cognition to reduce the job stress. This study builds a complete theoretical framework based on the job demand-control-support model, verifies the relationship among psychological contract, self-efficacy, job stress, and turnover intention, and takes the transformation leadership as a critical moderator that can strengthen or weaken the relationship between variables in the theoretical framework.

This study discusses the effect of self-efficacy and job stress on employee turnover intention from the positive and negative psychological cognition factors. The research results showed that self-efficacy has a negative impact on the employee turnover intention, and job stress has a strong negative impact on employee turnover intention. The findings are similar to the conclusions drawn by other scholars (De Simone et al., 2018; Afzal et al., 2019; Akgunduz and Gürel, 2019; Wen et al., 2020), that is, the positive aspect of self-efficacy and the negative aspect of job stress are important factors affecting employee turnover intention. This means, in the Chinese society in the pandemic, job stress drives employees to pursue a higher level of security; moreover, the conversion cost of turnover in the pandemic is relatively low, which drives employees to seek safer and more stable work environments. Thus, many uncertainties will indirectly increase the job stress during the COVID-19 pandemic, and employees will have a sense of inability to change working conditions because their negative moods cannot be vented, leading to the high turnover intention. However, self-efficacy is beneficial to reducing the employee turnover intention. As stated by scholars, self-efficacy makes employees more adaptable to organizational environments (De Simone et al., 2018; Etehadi and Karatepe, 2019) and takes a high level of organizational citizenship behaviors, and ensures employees' willingness to help others and remain in organizations despite of heavy workloads (Afzal et al., 2019; Khan et al., 2021). Different from job stress, self-efficacy represents the employees' cognition and belief in their own abilities. Uncertainties in work can be predicted precisely only through the grasp of expertise and the development of skills. Thus, employees with the high self-efficacy are able to master favorable conditions in the workplace to create opportunities and possibilities, leading to the reduction of turnover intention.

Furthermore, the research results also presented that self-efficacy has a negative impact on job stress, which is similar to the findings of other scholars (Sahin and Çetin, 2017; De Clercq et al., 2018). In other words, when employees are highly aware of and believe in their capabilities, they are more motivated to propose the solutions for work-related issues, and are more likely to have a sense of achievement in the process of achieving task goals and reducing the job stress. For individuals, challenging stress may be considered as a momentum to facilitate the self-growth inspire potential and achieve goals. But if there is no sufficient self-efficacy, the will and energy of individuals may be consumed virtually in face of overhigh stress factors such as time urgency and job demand, resulting in physical and mental fatigue and enhanced turnover intention.

The job demand-control-support model mainly discusses the job attitude and reaction of employees caused by job demand and job resources, and the resulting cognitive state, known as psychological contract, against organizations. The research results showed that the psychological contract has a negative impact on job stress and has a strong positive impact on the employee self-efficacy, which is similar to the arguments of other scholars (Hartmann and Rutherford, 2015; Kim et al., 2018b; Duran et al., 2019). This indicates that the psychological contract can be used as an effective indicator to test employees' identification with organizations and utilization of organizational resources (Liu et al., 2021). A high level of psychological contract enables employees to keenly perceive good job prospects provided by the organizations (Birtch et al., 2015), receive educational training, and skill training provided by the organization, thus improving the capabilities required to achieve goals and reducing stress arising from job uncertainties.

In order to understand the control degree of situational influence in the job demand-control-support model, this study assumes that the transformational leadership will moderate the direct relationship between variables. The research results showed that transformational leadership has a significant moderating effect on the relationships of psychological contract to job stress, self-efficacy to turnover intention, and job stress to turnover intention. Different from previous studies, transformational leadership in this study is used as a situational factor, but not an independent variable (Vasilaki, 2011). In addition to the effect of the transformational leadership context on the overall operation of the model, the development course from the stage when employees recognize the information conveyed by the transformational leadership to the social interaction and the sharing of goals with organizations (Vasilaki et al., 2016) can also be further discussed to offer valuable insights into discussions over turnover intention. Transformational leadership does not only represent the leadership of managers, but also can be considered as an important culture that is injected into organizations from top to bottom, which is conducive to disseminating and extending cultural elements and offering rich and deep understandings of leadership theories. This study concludes, the same as other scholars, that the leadership is not only the main source of support for employees (Brotheridge and Lee, 2003; Arnold and Walsh, 2015), but also the major stress source and primary cause of emotional exhaustion for employees (Carlson et al., 2012). Thus, incorporating leadership style into the research model will enrich the context and connotation of theories related to the organizational citizenship behaviors and human resources.

### Managerial Implications

In additional theoretical contributions, several management implications are also presented in this study. First of all, this study verifies that the psychological contract will affect many important outcomes of employees, and explain the reasons for many organizational behaviors or attitudes. For example, the psychological contract can intensely strengthen the self-efficacy, and affect the employee job stress. This reflects the important role of the psychological contract played in improving the overall employee efficacy. Second, this study demonstrates the effect of positive and negative psychological cognition (e.g., self-efficacy and job stress) on turnover intention. Third, this study also finds that the transformational leadership context in an organization is conducive to improving employees' psychological quality for adapting to work conditions, thus reducing the turnover intention. In other words, an appropriate job design can inspire employees' potential in controlling job stress through the technique diversity, information processing, and professionalism presented by their job, in addition to helping employees to understand and adapt to their job. Thus, this study makes the following suggestions: (1) The leadership should provide training courses about knowledge and skills required by employees to deal with a variety of businesses. The courses are better when designed to enhance employees' abilities to process information and solve problems independently, and to inspire their intention to retain in organizations and enhance the self-efficacy. (2) The leadership can extend the sense of job responsibilities vertically, and motivate employees to deeply understand the job implications, so as to build the job motivation and self-efficacy of employees and reducing the excessive negative mindset and turnover intention. (3) The leadership should offer more opportunities to get employees involved in decision making, ensure relevant rules and regulations can be implemented in a more fair and open manner, thus reducing the impact of uncertain contexts on employees and eliminating the possible resistance.

### Research Limitations

First, this study only takes the front-line staff of the service industry in the Chinese mainland, so whether the research results of this study can be generalized and inferred to other industries remains to be verified. Second, as this study uses the questionnaire survey to measure the cognition of respondents over variables in this study, the questionnaires used are a self-report that fails to measure the implicit attitudes of respondents, and it is likely to have the issue of common method variance. As a result, we suggest that researchers combine the scale and depth interview in future studies. Moreover, in this study, only front-line staff were sampled, but the managers/supervisors have not been surveyed. In this regard, the study suggests that subsequent researchers can add managers/supervisors to the questionnaire to conduct the cross-level hierarchical model analysis, so as to enrich the significance of practice.

Second, in terms of sampling, we suggest the combination of transformational leadership scale with the employees' evaluation for supervisor and self-evaluation of supervisors. Besides, it would be better to incorporate the general information such as length of service of the leadership, and paired samples of each team can also be collected to facilitate the extendibility of research findings, and make the research findings richer and more accurate. In addition, gender has been an important influencing factor for studies of organizational behaviors, because male employees may have a different grasp of contextual uncertainties from female employees. As a result, this study suggests considering the comparisons between men and women to offer richer and more valuable significance to the development of theoretical models.

Lastly, although a good result has been shown in this study by using the JDCS model, a western theoretical model, to discuss the employees' turnover intention in Chinese society in the COVID-19 pandemic, there are still many theoretical extensibilities. As such, we suggest the use of more diversified theoretical models in future studies to probe into attitudes and psychological states of employees in the Asian area or the Chinese cultural context.

## Data Availability Statement

The raw data supporting the conclusions of this article will be made available by the authors, without undue reservation.

## Ethics Statement

The studies involving human participants were reviewed and approved by Academic Committee of Jinan University. The patients/participants provided their written informed consent to participate in this study.

## Author Contributions

All authors contributed to the writing and method design and approved the submitted version.

## Conflict of Interest

The authors declare that the research was conducted in the absence of any commercial or financial relationships that could be construed as a potential conflict of interest.

## Publisher's Note

All claims expressed in this article are solely those of the authors and do not necessarily represent those of their affiliated organizations, or those of the publisher, the editors and the reviewers. Any product that may be evaluated in this article, or claim that may be made by its manufacturer, is not guaranteed or endorsed by the publisher.
